# Attenuation of Chronic Stress-Induced Depressive-like Symptoms by Fish Oil via Alleviating Neuroinflammation and Impaired Tryptophan Metabolism in Aging Rats

**DOI:** 10.1021/acs.jafc.3c01784

**Published:** 2023-09-28

**Authors:** Te-Hsuan Tung, Wen-De Lai, Hsiu-Chuan Lee, Kuan-Pin Su, Binar Panunggal, Shih-Yi Huang

**Affiliations:** † School of Nutrition and Health Sciences, Taipei Medical University, Taipei 110301, Taiwan; ‡ Department of Psychiatry & Mind-Body Interface Laboratory (MBI-Lab), China Medical University Hospital, Taichung 404018, Taiwan; § Department of Nutrition Science, Faculty of Medicine, Diponegoro University, Semarang 50275, Indonesia; ∥ Graduate Institute of Metabolism and Obesity Sciences, Taipei Medical University, Taipei 110301, Taiwan; ⊥ Nutrition Research Centre, Taipei Medical University Hospital, Taipei 110301, Taiwan; # College of Medicine, China Medical University, Taichung 404018, Taiwan; ¶ Center of Nutrition Research, Diponegoro University, Semarang 50275, Indonesia

**Keywords:** fish oil, geriatric depression, kynurenine pathway, glial cell, cognition

## Abstract

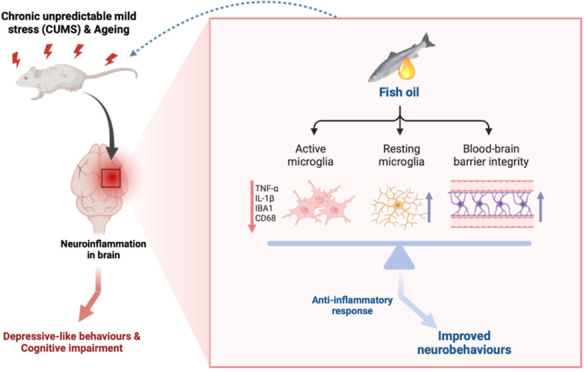

The prevalence of depression is increasing, and geriatric depression, in particular, is difficult to recognize and treat. Depression in older adults is often accompanied by neuroinflammation in the central nervous system (CNS). Neuroinflammation affects the brain’s physiological and immune functions through several pathways and induces depressive symptoms. This study investigated the relationship among depression, neuroinflammation, and fish oil supplementation. Thirty-six male Sprague–Dawley rats were used in an aging-related depression animal model to simulate geriatric depression. Cognitive function, depressive-like symptoms, peripheral nervous system and CNS inflammation status, and the tryptophan-related metabolic pathway were analyzed. The geriatric depression animal model was associated with depressive-like behaviors and cognitive impairment. The integrity of the blood–brain barrier was compromised, resulting in increased expression of ionized calcium-binding adapter molecule 1 and the glial fibrillary acidic protein in the brain, indicating increased neuroinflammation. Tryptophan metabolism was also negatively affected. The geriatric-depressive-like rats had high levels of neurotoxic 5-hydroxyindoleacetic acid and kynurenine in their hippocampus. Fish oil intake improved depressive-like symptoms and cognitive impairment, reduced proinflammatory cytokine expression, activated the brain’s glial cells, and increased the interleukin-10 level in the prefrontal cortex. Thus, fish oil intervention could ameliorate abnormal neurobehaviors and neuroinflammation and elevate the serotonin level in the hippocampus.

## Introduction

Depression is a common mental disorder. According to the 2017 Global Burden of Disease study conducted by the World Health Organization (WHO), approximately 264 million people experience severe depression globally.^1^ WHO data also revealed that approximately 700,000 people die by suicide every year. Geriatric depression or late-life depression refers specifically to the diagnosis of depression in patients of advanced age (over 60 years). The global prevalence rate of geriatric depression is approximately 13.3%. Studies have indicated that the economic burden on older adults with geriatric depression is 1.86 times that of average older adults.^2^ Untreated geriatric depression reduces the affected individuals’ quality of life and can even result in suicide.^2^ Compared with early onset depression, the geriatric type is associated with a lack of family history of depression and is strongly correlated with dementia, cerebrovascular disease, increased lateral ventricle volume, leukoencephalopathy, and resistance to late-onset depression drugs. Furthermore, the fatality rate is relatively high.^3^

Although several depression models with distinct advantages have been established, they cannot fully simulate general depression in humans, which is caused by countless environmental stressors. In the chronic unpredictable mild stress (CUMS) model developed by Katz et al., depression-like symptoms are induced in experimental animals by multiple environmental stressors.^4^ This simulation procedure is relatively consistent with the environmental triggers of human depression. The model has been widely used in pharmacological research of depression. Katz et al. observed that rodents had a preference for select sweet stimuli. After the rodents had been exposed to mild stress for several weeks, their preference was weaker or completely eliminated, reflecting the depression-related phenomenon of anhedonia. Consequently, elimination of the preference for sweet taste is used as an indicator of anhedonia. The preference for the sweet taste can be restored through treatment with tricyclic antidepressants, monoamine oxidase inhibitors, or atypical antidepressants.^5^

Previous studies comparing patients with depression versus healthy people found that lower blood levels of eicosapentaenoic acid and docosahexaenoic acid increased the synthesis of *n*-6 fatty acids and *n*-6 eicosanoids and interleukin (IL)-6, tumor necrosis factor (TNF)-α, and C-reactive protein concentrations.^6^ Studies have reported that insufficient dietary intake of *n*-3 polyunsaturated fatty acid (PUFA) is one cause of depression and is also associated with cardiovascular disease.^7,8^ The metabolism of serotonin has a significant correlation with depression. Preclinical and clinical studies have revealed that a long-term lack of dietary *n*-3 PUFA increases extracellular 5-hydroxyindoleacetic acid (5-HIAA) generation metabolized from serotonin and elevates the ratio of 5-HIAA/5-hydroxytryptamine (5-HT) in the brain.^9,10^ In addition, too much dietary arachidonic acid increases the levels of proinflammatory cytokines, and high concentrations of cytokines disrupt the metabolic pathway of 5-HT. Although the precise mechanism is unknown, IL-6 and 5-HIAA/5-HT are positively correlated in patients with depression.^7,11^ Hence, in this study, we investigated whether chronic stress impacts neuroinflammation and the tryptophan metabolic pathway in a geriatric depression animal model and explored the effects of fish oil intervention.

## Materials and Methods

### Materials, Diets, and Reagents

Soybean oil (Taiwan Sugar Co., Tainan City, Taiwan) and corn oil (God Bene Enterprises Co., Yunlin County, Taiwan) were purchased from a local supermarket. Fish oil was purchased from Chueh Hsin Co. Ltd. (New Taipei City, Taiwan). The components of the test diets were based on the American Institute of Nutrition (AIN)-93 M diet with 4% (w/w) oil, which provided the distinct fatty acids required for this study. The standard diet consisted of 4% (w/w) soybean oil; the fish oil diet consisted of 2% fish oil and 2% soybean oil; and the corn oil diet consisted of 2% corn oil and 2% soybean oil. The soybean and corn oils were purchased from a local supermarket. All chemicals used in this study were obtained from Sigma (St. Louis, MO, USA).

### Animals and Experimental Design

For our animal model, we used 36 male Sprague–Dawley rats (6 weeks old) obtained from the BioLASCO animal facility in Taiwan. The rats were housed under controlled environmental conditions [temperature: 22 ± 2 °C, humidity: 60%, and a 12 h light–dark cycle (light from 08:00 to 20:00)]. The study was conducted in accordance with institutional guidelines, and the study protocol was approved by the Taipei Medical University Institutional Animal Care and Use Committee (permit number: LAC-20160405).

After a 2 week acclimation period, the rats were divided into the following six groups: the control (C) group, “D-gal-induced aging” (A) group, “D-gal-induced aging with CUMS” (AS) group, “D-gal-induced aging with CUMS and supplemented fish oil diet” (FAS) group, “D-gal-induced aging with CUMS and supplemented with corn oil diet” (CAS) group, and “D-gal-induced aging with CUMS treated with imipramine medicine” (MAS) group. The fatty acid compositions of the oils and the diet composition were modified on the basis of the AIN-93 M formula, which is listed in the Supporting Information Tables S1 and S2. Food and water were provided ad libitum. The rats’ food intake and body weight were recorded during the experiment.

**Figure 1:**
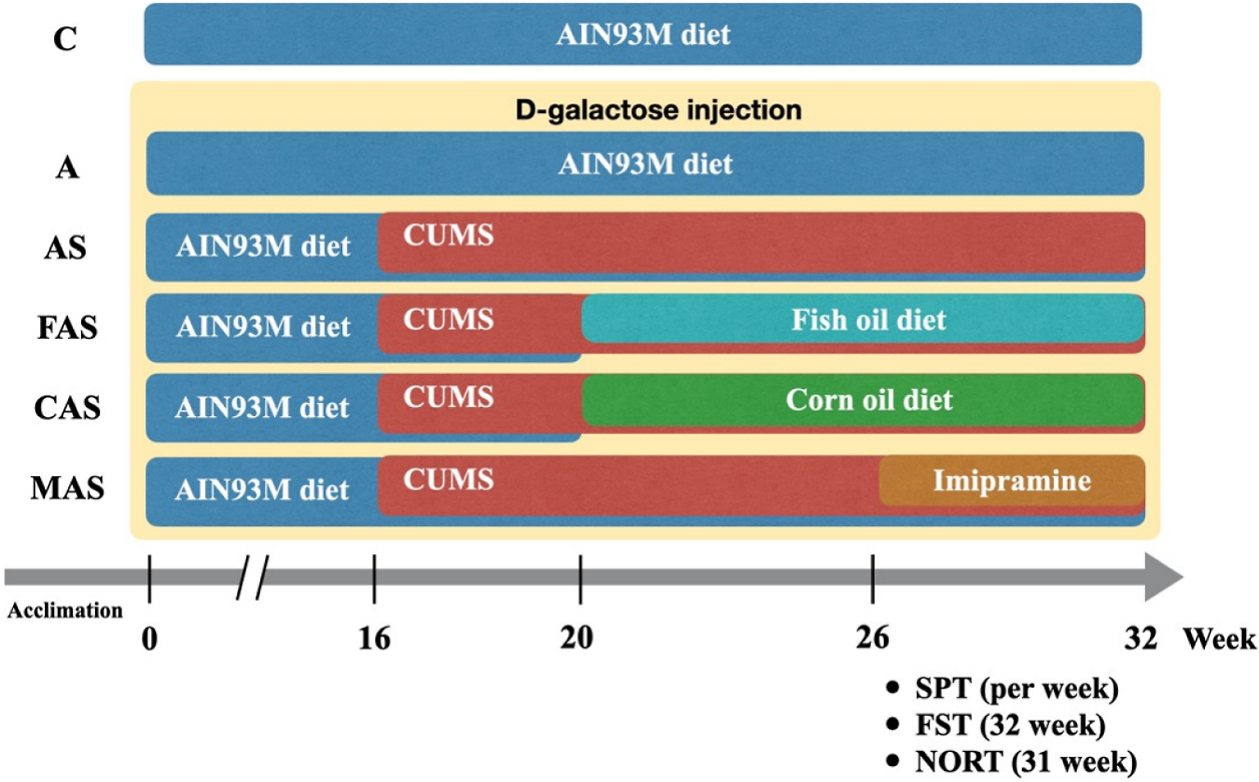
Flow diagram of the d-galactose-induced aging protocol. Rats were divided into six groups. *n* = 6. CUMS; SPT; FST; NORT; C, control group; A, induced aging group; AS, induced aging + CUMS stimulation group; FAS, fish oil + AS group; CAS, corn oil + AS group; MAS, imipramine medicine + AS group.

The experiment lasted for 32 weeks. We first began to induce aging. With the exception of the C group, the groups were administered subcutaneous d-galactose (D-gal) injections (600 mg/kg of body weight). CUMS stimulation was performed to induce symptoms of melancholia in week 16, and the sucrose preference test (SPT) was performed to monitor behavioral changes. A dietary intervention was initiated in week 20, that is, after 4 weeks of the rodents being exposed to stress (Figure 1). The FAS and CAS groups followed their respective fish oil and corn oil diets from week 20 until the end of the experiment. Imipramine (Sigma-Aldrich, Burlington, MA, USA) was dissolved in the MAS group’s drinking water (20 mg/kg) daily from week 26 to week 32. The experiments were terminated when we observed that the FAS group had experienced anhedonia symptoms for 2 weeks. The novel object recognition test (NORT) was performed in week 31. Each rat was subjected to the forced swim test (FST), after which the rats were immediately anesthetized and sacrificed.

### CUMS Model

The primary purpose of the stress induced in this model was to cause psychological stress rather than physical pain in rats to simulate the process of depression in humans. The research method was determined in reference to the literature^12–14^ and was adjusted in accordance with the laboratory environment. The effectiveness of the CUMS model was determined through the SPT. The methods for inducing stress were as follows: a 30° cage tilt, food and water deprivation, damp sawdust (250 mL of water poured on the sawdust bedding), a lack of sawdust bedding, reversal of circadian rhythm, restricted activity, compressed living space, social stress, and cold swimming (10 °C). The same stressor was not applied on consecutive days.^15,16^

### Behavioral Tests

For the SPT, we prepared two water bottles; one bottle contained normal distilled water and the other contained 1% (w/w) sucrose water. The experiment was conducted for a total of 12 h once per week. Prior to the test, the rats fasted for 24 h to increase their willingness to ingest liquid during the trial. The sucrose water bottle was placed in different positions each week to prevent the animals from relying on their memorization of the location of the water bottle.

The FST was conducted to detect the helplessness of the rats when they were faced with acute stress. The experiment was conducted in two stages. In the pretest stage, we prepared a cylindrical acrylic water tank with a diameter of 35 cm, a height of 70 cm, and a water depth of 55 cm. A 15 min pre-experiment was conducted to ensure that the rats would not escape from the water tank. The rats were then dried and returned to their cage. After 24 h, a probe test was conducted for 6 min. The behavior of the rats throughout the whole process was recorded using a video camera and was analyzed using Forced SwimScan version 2.0 (CleverSys, Reston, VA, USA). The following behavioral definitions were used in this study: (1) violent struggling period: vigorous swimming with limbs slapping the water surface, climbing the edge of a cylinder, and diving. (2) Small action swimming: slow water swimming to maintain body balance and to keep the head above the water. (3) Immobile time: floating on the water surface without performing other actions.

In the experiment, the NORT was used to determine the working memory function. A black box with a length of 50 cm, a width of 50 cm, and a height of 30 cm was used. The experiment was divided into three stages. The first stage was the habituation stage. Each rat was placed in the black box for 10 min and allowed to explore freely, after which it was returned to its cage. A 75% alcohol solution was used to clean the box between each experiment. The second stage was the training stage. We prepared two identical pepper pots with a height of 6.8 cm and a diameter of 5 cm and drew an area measuring 20 cm in length and 20 cm in width in the middle of the box. After the rat was placed in the corner of the box, the rat could explore freely for 10 min. After each experiment, the inside of the box and the body of the pepper pot were wiped clean with 75% alcohol solution to prevent the residual flavor from being disturbed. The third stage was the testing stage. We prepared a pepper pot as in the previous stage and arranged the other LEGO bricks with a length of 6.4 cm, a width of 3 cm, and a height of 5.5 cm. We placed two different objects in the diagonal part of the divided area in the box. Each rat was allowed to explore for 10 min, and the inside of the box and the body of the pepper pot were wiped clean using alcohol solution at the end of each experiment to prevent any residual flavor interference. The analysis was conducted by trained professionals. The analysis method consisted of observing for how long the rats explored the two objects. The rats were not considered to have explored the object if they just climbed and sat on the object without inspecting it. An exploration time of 20 s or longer was counted as successful exploration. Finally, we calculated the preference index for analysis. The formula is as follows^17^
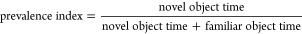


### Examination of Biochemical and Inflammatory Parameters

Aspartate aminotransferase (AST) and alanine aminotransferase (ALT) were measured by nicotinamide adenine dinucleotide enzymatic assay. Total cholesterol (TC) was examined using enzymatic colorimetric method. Total glyceride (TG) was measured by glycerol-3-phosphate oxidase method. Low-density lipoprotein (LDL-c) was examined using liquid selective detergent assay. High-density lipoprotein (HDL-c) was examined using accelerator selective detergent assay. Blood urine nitrogen (BUN) was measured by urease enzymatic assay. Biochemistry parameters [8-hydroxy-2′-deoxyguanosine (8-OHdG), advanced glycation end products (AGEs), and malondialdehyde (MDA)] and proinflammatory indices (TNF-α, IL 1 beta [IL-1β], and IL-6) were measured in plasma and hippocampus samples, respectively. Plasma 8-OHdG was examined using a DNA/RNA oxidative damage enzyme-linked immunosorbent assay (ELISA) kit (589320, Cayman Chemical, Ann Arbor, MI, USA), plasma AGEs were examined using the OxiSelect AGE Competitive ELISA kit (STA-817, Cell Biolabs, San Diego, CA, USA), and MDA was examined using colorimetric 2-thiobarbituric acid reactive substances (TBARS) assay. The hippocampus TNF-α level was examined using a rat TNF-α ELISA kit (438207, BioLegend, San Diego, CA, USA) in accordance with the manufacturer’s instructions. Plasma IL-1β and IL-6 levels were determined using a rat IL-1β Quantikine ELISA kit (RLB00, R&D Systems, Minneapolis, MN, USA) and a rat IL-6 Quantikine ELISA kit (R6000B, R&D Systems, Minneapolis, MN, USA), respectively.

### Gut Section Hematoxylin and Eosin Staining

After sacrificing, we cut 0.5 cm of colon samples from the anus, fixed them in formalin, and then embedded them in paraffin blocks. Next, 4 μm tissue sections were cut and stained with hematoxylin and eosin (H&E) for histological analysis under a light microscope (DM2700 M, Leica Microsystems, Wetzlar, Germany). Tissues were scored for inflammation, edema, goblet cell depletion, and epithelial damage in accordance with the following criteria: 0 = none present, 1 = minimal change, 2 = mild change, 3 = moderate change, and 4 = severe change. The scores for inflammation, edema, goblet cell depletion, and epithelial damage were summed to obtain sum colon scores. The analysis was conducted by trained professionals. Total scores for colitis (the total colitis index) were then added, resulting in a combined histologic score ranging from 0 to 16.^18^

### Kynurenine Pathway Analysis

The analysis procedure followed that described in the literature.^19^ In brief, the hippocampus tissue samples were homogenized using deionized water. We then transferred the supernatant to the following analysis. The Agilent 6470 triple quadrupole liquid chromatography–mass spectrometer/mass spectrometer system was used. The chromatographic separation was achieved on an ACQUITY UPLC HSS T3 Column, 100 Å, 1.8 μm, 2.1 × 100 mm (Waters Co., Milford, MA, US) at 40 °C. The mobile phase consisted of (A) water with 0.1% formic acid (FA) and (B) methanol with 0.1% FA at a flow rate of 0.4 mL/min. The gradient elution was programmed as follows: 0–8 min, 50% A; 8–8.1 min, 50–0% A; 8.1–10 min, 0% A; 10–10.1 min, 0–100% A; 10.1–12 min, 100% A.

The mass spectrometric detection was performed using multiple reaction monitoring with an electrospray ionization source in positive mode. Ion source parameters were optimized with the isocratic mobile phase composition without column separation. The conditions were as follows: ion spray voltage, 5000 V; temperature, 300 °C; Sheath gas flow, 11 psi; nebulizer gas, 45 psi; and heater gas, 40 psi. Data acquisition and processing were performed with the Agilent MassHunter Workstation Software. The standard solutions of 5-HIAA, tryptophan, kynurenine, kynurenic acid (KA), and 3-hydroxykynurenine (3-HK) were purchased from Sigma-Aldrich (Burlington, MA, US).

### Western Blotting

The rat prefrontal cortex tissue samples were prepared in RIPA buffer (RIP001, Bioman Scientific, New Taipei, Taiwan) supplemented with a protease and phosphatase inhibitor cocktail (P8340, Sigma-Aldrich) and were centrifuged for 15 min at 12,000*g*. The supernatant was prepared and processed using the sodium dodecyl sulfate–polyacrylamide gel electrophoresis system. Then, the samples were transferred to polyvinylidene difluoride membranes. All membranes were blocked using 5% bovine serum albumin solution, and primary antibody incubation was performed overnight at 2–8 °C. The next day, the membranes were incubated with secondary antibody for 1 h and visualized using the UVP BioSpectrumAC Imaging System. The primary antibodies used were zonula occludens-1 (ZO-1) (1:1000, 21773-1-AP, Proteintech Group, Inc., Rosemont, IL, US), occludin (1:1000, 13409-1-AP, Proteintech Group, Inc., Rosemont, IL, US), inducible nitric oxide synthase (iNOS) (1:1000, 18985-1-AP, Proteintech Group, Inc., Rosemont, IL, US), glial fibrillary acidic protein (GFAP) (1:2000, 16825-1-AP, Proteintech Group, Inc., Rosemont, IL, US), ionized calcium-binding adapter molecule 1 (IBA1) (1:500, 10904-1-AP, Proteintech Group, Inc., Rosemont, IL, US), IL-10 (1:500, 20850-1-AP, Proteintech Group, Inc., Rosemont, IL, US), and β-actin (1:5000, AF7018, Affinity Biosciences, Cincinnati, OH, US).

### Histological Staining

Immunohistochemistry (IHC) analysis was performed in accordance with R&D System’s protocol. In brief, brain samples were resected after the rats had been sacrificed, and the samples were snap-frozen using liquid nitrogen for 15 s. The frozen brain samples were stored until cryosectioning was performed using the Leica Biosystems Cryostat Microtome (CM3050S, Wetzlar, Germany); the thickness of each section was 10 μm. The sections were fixed using 10% formalin for 8 min at 4 °C and were then submerged in heated antigen retrieval solution for 5 min (TE buffer, pH = 9.0). Next, the sections were incubated with 3% H_2_O_2_ solution and blocking buffer, and primary antibody incubation was performed overnight at 2–8 °C. On the second day, the samples were incubated using the secondary antibody reagent for 60 min. We used diluted DAB chromogen solution to visualize the results. The slides were observed by using a microscope (Leica Microsystems, Wetzlar, Germany). The primary antibodies employed were IBA1 (1:100, 10904-1-AP, Proteintech Group, Inc., Rosemont, IL, USA) and CD68 (1:100, 28058-1-AP, Proteintech Group, Inc., Rosemont, IL, US).

### Statistical Analysis

All data are expressed as the mean ± standard error of the mean (SEM) or the standard deviation (SD). Prism 9.1.1 (GraphPad Software, La Jolla, CA, USA) was used to perform the analyses. We employed one-way analysis of variance (ANOVA) followed with Tukey’s *posthoc* test in the analyses. ImageJ software version 1.53.11 was used to analyze the Western blot data and IHC results. Significant differences were indicated by *p* < 0.05.

## Results

### Basic Biochemistry Analysis Cytokines and Oxidative Stress in Plasma

Administering d-galactose elevated TNF-α and IL-1β levels (A group), and CUMS stimulation did not worsen them (AS group). Fish oil diet (FAS group) decreased the levels of TNF-α and IL-1β. But there was no difference in IL-6 levels in plasma. Essential indicators of the effectiveness of the D-gal-induced aging model were 8-OHdG, AGEs, and oxidative stress. AGE concentrations increased in A and AS groups after D-gal injection. 8-OHdG, a potent indicator of aging, was substantially higher in all treatment groups than in the C group. Results indicated the successful induction of aging by using the mimetic aging model. To measure plasma oxidative stress, a TBARS assay was used to assess MDA levels. A, AS, and CAS groups had higher levels of MDA than did the C group. MDA levels were restored in MAS and FAS groups, indicating higher levels of lipid oxidative stress in A, AS, and CAS groups (Table 1).

**Table 1:** Basic Biochemistry Analysis Data in Plasma^a^

	groups						
item	C	A	AS	FAS	CAS	MAS	*p* value
ALB (g/dL)	4.09 ± 0.06^ns^	4.05 ± 0.09^ns^	4.02 ± 0.07^ns^	4.07 ± 0.04^ns^	4.01 ± 0.07^ns^	4.08 ± 0.07^ns^	0.2628
TG (mg/dL)	80.0 ± 11.9^b^	130.1 ± 34.0^a^	115.5 ± 25.1^ab^	86.7 ± 40.8^ab^	96.0 ± 19.4^ab^	81.3 ± 12.0^b^	0.0113
TC (mg/dL)	63.5 ± 7.0^ns^	65.2 ± 12.4^ns^	63.3 ± 13.0^ns^	55.7 ± 6.3^ns^	62.7 ± 16.3^ns^	75.8 ± 5.8^ns^	0.0867
LDL-c (mg/dL)	8.43 ± 3.99^b^	20.3 ± 9.5^a^	28.3 ± 8.4^a^	20.2 ± 4.6^a^	24.3 ± 4.6^a^	20.7 ± 7.0^a^	0.0002
HDL-c (mg/dL)	25.2 ± 4.3^a^	16.2 ± 4.7^b^	16.0 ± 2.2^b^	19.5 ± 2.5^ab^	19.7 ± 2.7^ab^	24.2 ± 3.4^a^	<0.0001
ALT (U/L)	32.1 ± 13.2^ns^	71.5 ± 42.2^ns^	52.1 ± 59.2^ns^	32.4 ± 7.2^ns^	32.0 ± 5.4^ns^	25.0 ± 4.7^ns^	0.1077
AST (U/L)	134 ± 49^b^	262 ± 119^ab^	378 ± 98^a^	302 ± 57^a^	263 ± 84^ab^	320 ± 114^a^	0.0022
BUN (mg/dL)	20.6 ± 1.5^ns^	19.9 ± 0.9^ns^	20.5 ± 0.6^ns^	18.5 ± 1.6^ns^	19.2 ± 0.6^ns^	19.1 ± 2.2^ns^	0.0736
TNF-α (pg/mL)	41.7 ± 2.3^b^	56.6 ± 6.1^a^	69.0 ± 3.2^a^	42.8 ± 5.9^b^	54.5 ± 2.9^a^	51.4 ± 3.4^a^	0.0150
IL-1β (pg/mL)	20.8 ± 3.4^c^	49.2 ± 7.2^a^	49.0 ± 5.6^a^	34.3 ± 3.9^b^	43.8 ± 4.5^a^	28.1 ± 3.2^bc^	0.0002
IL-6 (pg/mL)	40.1 ± 2.3^ns^	41.5 ± 2.0^ns^	41.3 ± 2.9^ns^	40.0 ± 2.7^ns^	39.8 ± 5.3^ns^	38.5 ± 2.4^ns^	0.6036
corticosteroid (ng/mL)	131 ± 20^b^	217 ± 30^a^	208 ± 28^a^	195 ± 21^a^	201 ± 23^a^	190 ± 32^a^	<0.0001
8-OHdG (pg/mL)	224 ± 98^b^	809 ± 339^a^	792 ± 185^a^	791 ± 378^a^	797 ± 244^a^	976 ± 414^a^	0.0034
AGE (μg/mL)	15.0 ± 3.59^b^	26.5 ± 10.1^a^	28.2 ± 6.92^a^	21.1 ± 4.69^ab^	23.4 ± 4.49^ab^	19.1 ± 6.27^ab^	0.0131
MDA (μM/100 mL)	20.0 ± 1.8^b^	35.7 ± 7.3^a^	37.5 ± 12.4^a^	21.7 ± 6.7^b^	41.2 ± 8.0^a^	22.9 ± 6.4^b^	<0.0001

a Values are presented as the mean ± SD (*n* = 6). Different subscript letters^(a.b.c)^ indicate significant differences among groups at *p* < 0.05. ^ns^ = no significant difference. ALB, albumin; TG, plasma triglyceride; TC, total cholesterol; LDLc, low-density lipoprotein cholesterol; HDL-c, high-density lipoprotein cholesterol; ALT, alanine aminotransferase; AST, aspartate aminotransferase; BUN, blood urea nitrogen; TNF-α, tumor necrosis factor-alpha; IL-1β, interleukin-1 beta; IL-6, interleukin-6; AGE, advanced glycation end product; 8-OHdG, 8-Oxo-deoxyguanosine; MDA, malodialdehyde; C, control group; A, induced aging group; AS, induced aging + CUMS stimulus group; FAS, fish oil + AS group; CAS, corn oil + AS group; MAS, medicine + AS group.

### Depressive-like Behavior and Cognitive Function Evaluation

**Figure 2:**
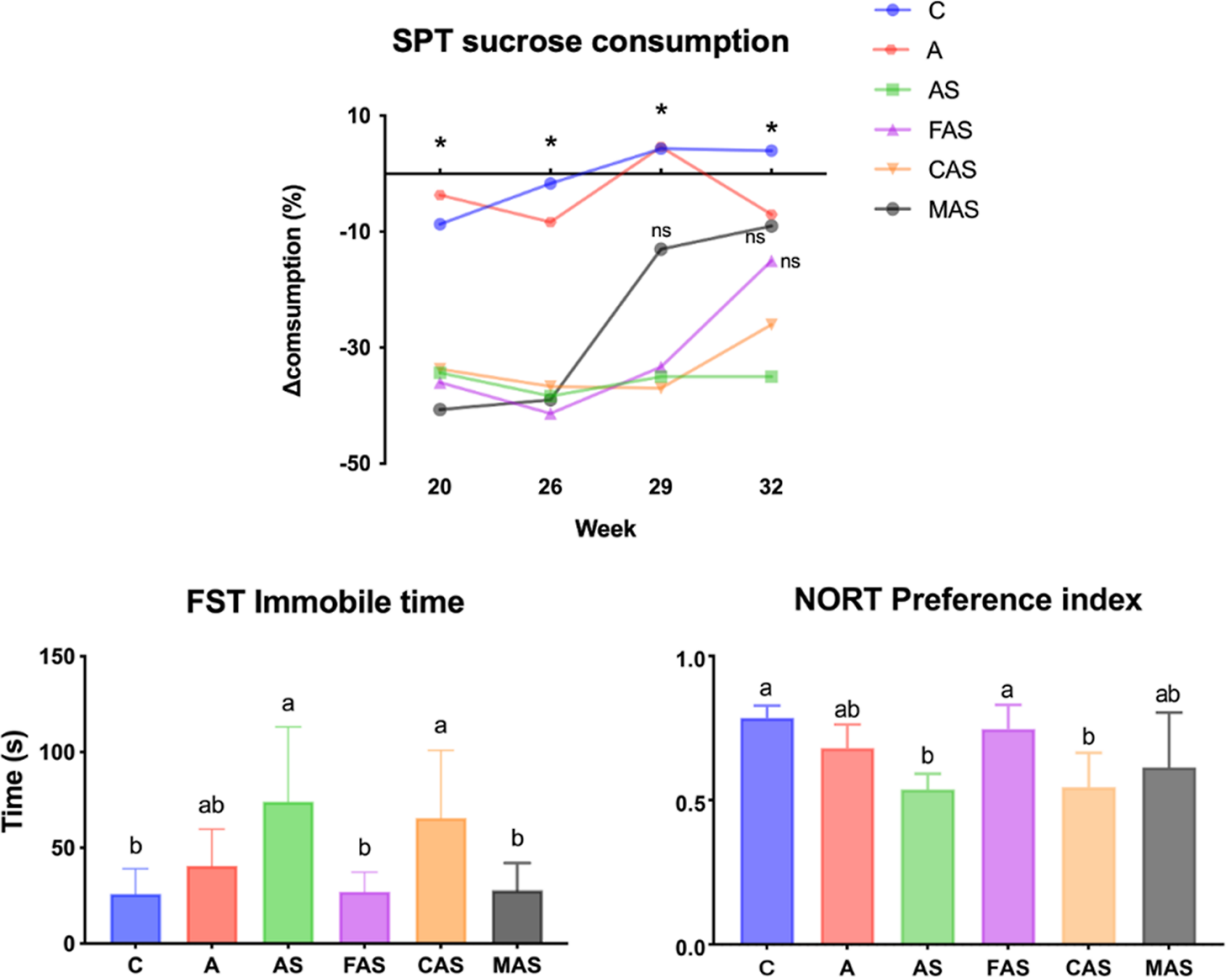
Depressive-like behavior tests and cognitive function results. Values are presented as mean ± SD. *n* = 6. ANOVA, SPT *p* < 0.0001; FST, *p* = 0.003; NORT, *p* < 0.0001. ^ns^ mean no significant difference compared with the C group. * Mean significant differences between C group and other groups (except the A group and the groups marked ^ns^). Different subscript letters^(a.b)^ indicate significant differences between groups (*p* < 0.05). SPT, sucrose preference test; FST, force swimming test; NORT, novel object recognition test; C, control group; A, induced aging group; AS, induced aging + CUMS stimulation group; FAS, fish oil + AS group; CAS, corn oil + AS group; MAS, imipramine medicine + AS group.

The SPT is a practical behavioral test used to monitor and evaluate the effectiveness of CUMS stimulation. CUMS stimulation was conducted from week 16, and the SPT was implemented from week 15 to train the rodents (data not shown). One month of CUMS stimulation caused significantly lower sucrose consumption in AS, FAS, CAS, and MAS groups. These anhedonia symptoms were ameliorated by the imipramine treatment from week 29 and the fish oil intervention ameliorated the anhedonia from week 31. By contrast, the corn oil intervention had no effect on depressive-like behavior outcomes in the SPT (Figure 2).

The FST showed that the immobility time of the AS and CAS groups was significantly longer than that of the C group, indicating helplessness-related behavior. The A group had a similar immobility time. The FAS and MAS groups had smaller immobility times than the AS group, indicating that fish oil and imipramine can improve helplessness-related behaviors. The NORT preference index result showed that D-gal combined with CUMS stimulation (the AS group) impaired working memory function, while fish oil diet intake improved cognitive function. Otherwise, the corn oil had no effect on the NORT results (Figure 2).

### H&E Staining of Colon and IHC of Hippocampal Tissue Cryosections

**Figure 3:**
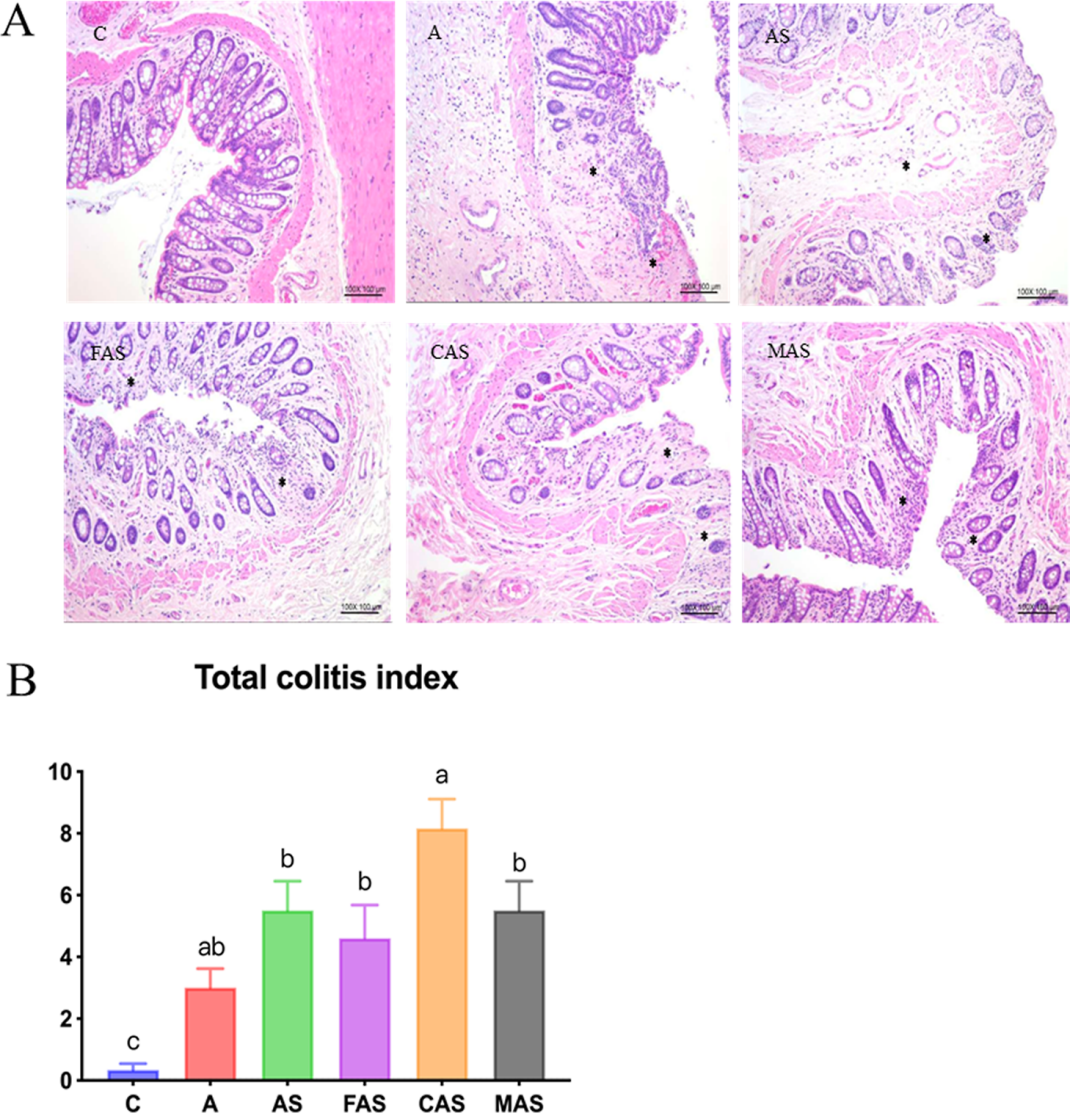
Colitis analysis of colon H&E-stained sections: (A) C group with normal colon. In the A, AS, FAS, and CAS groups, the asterisks indicate inflammatory cell infiltration. In the MAS group, the asterisk indicates mild focal epithelial damage. (B) Statistical analysis of the total colitis index of all groups. Values are expressed as the mean ± SEM. *n* = 6. ANOVA, *p* < 0.0001. Different subscript letters^(a.b.c)^ indicate significant differences between groups (*p* < 0.05).

To determine the colitis score, we assessed minimal to mild focal mucosal architectural abnormalities, minimal focal ulceration, mild diffuse crypt dilation, aberrant crypt foci, crypt loss, distortion of mucosal glands, and focal epithelial degeneration, all of which were observed to be more prevalent in all treatment groups than in the C group. The CAS group had the highest colitis score in this analysis (Figure 3).

**Figure 4:**
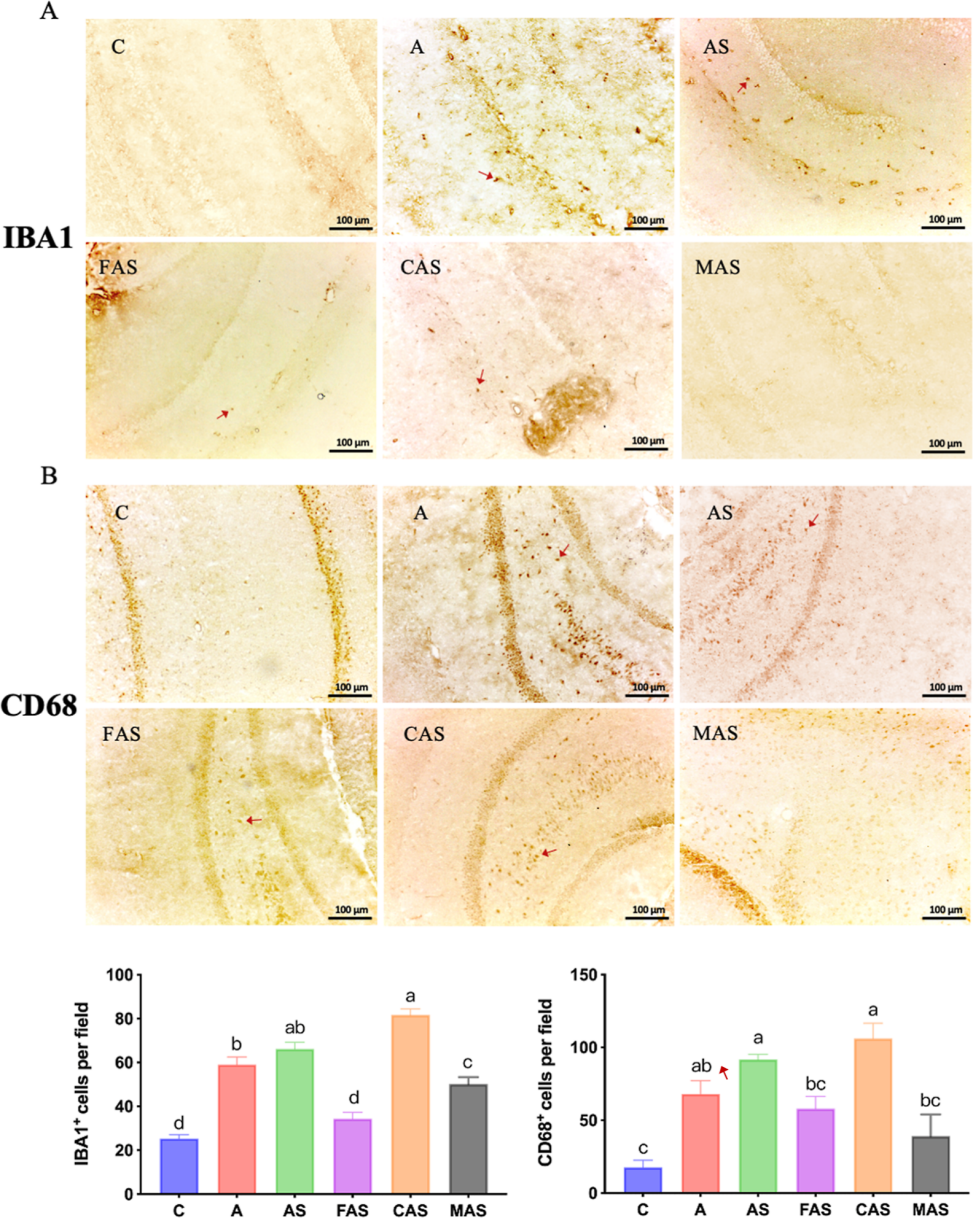
IHC analysis of hippocampus tissue. (A) IBA1 and (B) CD68 expression in the hippocampus in each group. Arrows represent target protein expression. Values are presented as the mean ± SD *n* = 6. ANOVA, IBA1, and CD68, *p* < 0.0001. Different subscript letters^(a.b)^ indicate significant differences between groups (*p* < 0.05). C, control group; A, induced aging group; AS, induced aging + CUMS stimulus group; FAS, fish oil + AS group; CAS, corn oil + AS group; MAS, imipramine medicine + AS group.

IHC analysis can illustrate the presence of a target protein in a specific tissue area. In this study, we used a cryostat microtome to perform cryosection of brain tissue and to detect the pro-inflammatory-type microglia markers IBA1 and CD68. Results showed increased IBA1 expression in the A and AS groups due to D-gal-induced aging and CUMS stimulation. Imipramine and fish oil had beneficial effects, while corn oil had a detrimental effect. In this study, the expression of CD68 was considerably higher (due to induced aging and CUMS stimulation) in the A and AS groups than in the C group. Imipramine treatment and the fish oil intervention ameliorated neuroinflammation, whereas the corn oil intervention had no effect (Figure 4B).

### Hippocampal Inflammatory Parameters

Inflammatory cytokine quantities in brain tissue were evaluated using an ELISA kit. Results indicated an increase in IL-1β levels in the A and AS groups, attributable to D-gal-induced aging. Imipramine administration and fish oil dietary intervention led to a significant decrease in IL-1β levels. D-gal administration and CUMS stimulation did not significantly elevate IL-6 levels in the hippocampus; the intervention of the corn oil diet exacerbated the inflammatory condition by increasing IL-6 levels. There was no significant difference in TNF-α levels in the hippocampus between the C and A groups, but CUMS stimulation and a corn oil diet further increased TNF-α levels in comparison to the C group (Table 2).

**Table 2:** Pro-Inflammatory Cytokines and TRYCAT Analysis in the Hippocampus^a^

	groups						
item	C	A	AS	FAS	CAS	MAS	*p* value
Cytokines							
IL-1β (pg/mg protein)	1.17 ± 0.21^b^	2.23 ± 0.84^a^	2.17 ± 0.49^a^	0.95 ± 0.50^b^	1.53 ± 0.50^ab^	1.17 ± 0.31^b^	0.006
IL-6 (pg/mg protein)	4.86 ± 0.93^b^	5.23 ± 0.91^ab^	6.30 ± 0.85^ab^	5.09 ± 0.80^ab^	6.82 ± 1.55^a^	5.47 ± 1.01^ab^	0.018
TNF-α (pg/mg protein)	3.23 ± 0.78^b^	4.15 ± 0.36^ab^	4.48 ± 0.50^a^	3.63 ± 1.24^ab^	4.50 ± 0.51^a^	3.52 ± 0.49^ab^	0.015
TRYCAT							
serotonin (ng/mg tissue)	0.38 ± 0.11^b^	0.21 ± 0.05^bc^	0.12 ± 0.06^c^	0.42 ± 0.12^ab^	0.20 ± 0.04^bc^	0.75 ± 0.39^a^	<0.001
5-HIAA (pg/mg tissue)	3.98 ± 0.66^b^	7.11 ± 1.53^a^	7.61 ± 1.01^a^	3.16 ± 1.10^b^	6.95 ± 2.28^a^	3.47 ± 1.22^b^	<0.001
5-HIAA/5-HT	0.012 ± 0.005^b^	0.034 ± 0.013^b^	0.085 ± 0.039^a^	0.008 ± 0.003^b^	0.033 ± 0.009^b^	0.005 ± 0.003^b^	<0.001
tryptophan (pg/mg tissue)	45.5 ± 12.0^ns^	44.5 ± 12.0^ns^	30.0 ± 7.50^ns^	38.0 ± 8.50^ns^	23.5 ± 14.0^ns^	39.5 ± 29.5^ns^	0.147
kynurenine (pg/mg tissue)	3.56 ± 0.37^b^	3.96 ± 0.92^ab^	5.80 ± 1.80^a^	4.36 ± 0.72^ab^	5.70 ± 0.94^a^	5.20 ± 1.71^ab^	0.009
KYN/TRP	0.08 ± 0.02^b^	0.10 ± 0.04^b^	0.20 ± 0.06^a^	0.19 ± 0.02^a^	0.25 ± 0.10^a^	0.23 ± 0.15^a^	0.004
KA (pg/mg tissue)	2.15 ± 0.12^b^	2.32 ± 0.25^ab^	2.71 ± 0.40^a^	2.20 ± 0.21^b^	2.47 ± 0.34^ab^	2.37 ± 0.24^ab^	0.018
3-HK (pg/mg tissue)	0.14 ± 0.02^ns^	0.13 ± 0.02^ns^	0.15 ± 0.03^ns^	0.16 ± 0.02^ns^	0.16 ± 0.03^ns^	0.15 ± 0.02^ns^	0.236

a Values are presented as the mean ± SD (*n* = 6). ns = no significant difference. Different subscript letters^(a.b.c)^ indicated significant differences among groups at *p* < 0.05. ^ns^ = no significant difference; IL-1β, interleukin-1β; IL-6, interleukin-6; TNF-α, tumor necrosis factor-α; TRYCAT, tryptophan catabolites; 5-HT, serotonin; 5-HIAA, 5-Hydroxyindoleacetic acid; KYN, kynurenine; TRP, tryptophan; KA, kynurenic acid; 3-HK, 3-hydroxykynurenine; C, control group; A, induced aging group; AS, induced aging + CUMS stimulus group; FAS, fish oil + AS group; CAS, corn oil + AS group; MAS, medicine + AS group.

### Tryptophan-Related Pathway Catabolite Quantitative Analysis

In the hippocampus, we observed that the serotonin level was reduced through induced aging and CUMS stimulation in the AS group. Imipramine (MAS group) and fish oil diet (FAS group) restored the serotonin level. Tryptophan-related catabolites, including 5-HIAA and the primary serotonin metabolite, were higher with aging and CUMS. Imipramine and fish oil lowered the 5-HIAA concentration, but tryptophan level did not differ. Kynurenine, the first kynurenine pathway metabolite, was increased with CUMS, with no difference for the A group. FAS and MAS groups had slightly reduced kynurenine levels. Kynurenine is metabolized into 3-HK or KA. KA level, elevated through induced aging and CUMS stimulation, was restored slightly through imipramine treatment and fish oil and corn oil intake. At last, the 3-HK level did not differ between the groups (Table 2).

### Protein Expression Analysis of Prefrontal Cortex Tissue

**Figure 5:**
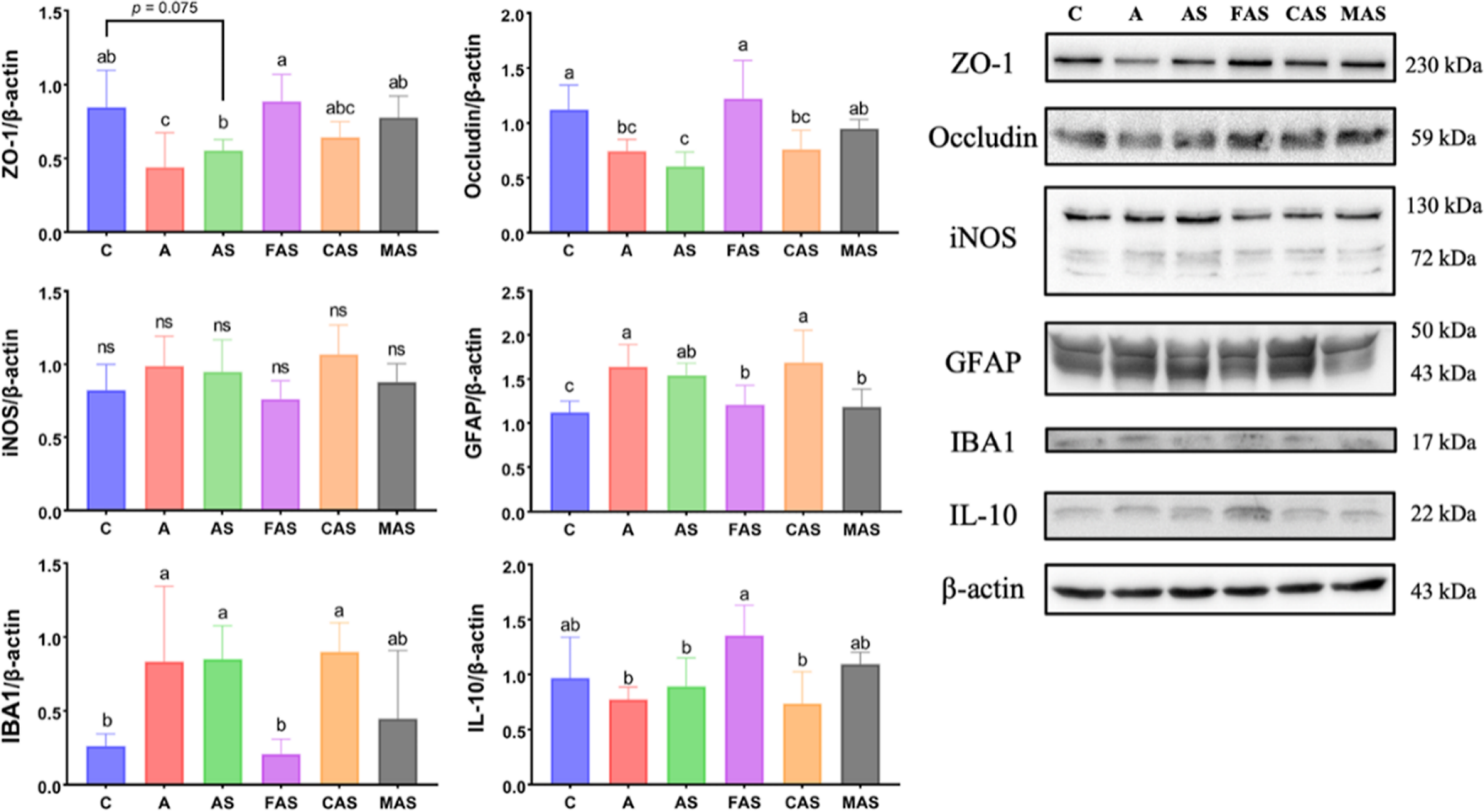
Western blot results for prefrontal cortex tissue. Values are presented as the mean ± SD *n* = 6. ns = no significant difference. ANOVA, ZO-1, *p* = 0.0007; occludin, *p* < 0.0001; iNOS, *p* = 0.067; GFAP, *p* = 0.0002; IBA1, *p* = 0.0005; IL-10, *p* = 0.0022. Different subscript letters^(a.b.c)^ indicate significant differences between groups (*p* < 0.05). *n* = 6. ZO-1, zonula occludens-1; iNOS, inducible nitric oxide synthase; GFAP, glial fibrillary acidic protein; IBA1, ionized calcium-binding adapter molecule 1; IL-10, interleukin-10; C, control group; A, induced aging group; AS, induced aging + CUMS stimulus group; FAS, fish oil + AS group; CAS, corn oil + AS group; MAS, imipramine medicine + AS group.

We observed reduced expression of ZO-1 and occludin in the A and AS groups (with the astrogliosis marker GFAP and activated microglia marker IBA1 present). The FAS group had higher expression of both proteins than the A group, and the MAS group had higher occludin expression than the AS group. Fish oil intervention was beneficial, ameliorating GFAP and IBA1 overexpression and elevating the IL-10 concentration. However, the iNOS protein expression in prefrontal cortex tissue did not differ between any groups. However, iNOS protein expression, the oxidative stress indicator, in the prefrontal cortex tissue did not differ between the groups (Figure 5).

## Discussion

Little research has explored geriatric depression using both preclinical studies and clinical trials. One challenge is creating animal models that simultaneously reflect aging-related and depression-related factors simultaneously. During the experiment, half of the dietary lipids were replaced by fish oil or corn oil to evaluate the effect of n-3 PUFAs on depressive-like aging status and neurodegenerative behavioral outcomes. Studies showed that D-gal can induce senescence in vivo, leading to oxidative stress, neuroinflammation, and cognitive deficits.^20,21^ Unchallenged natural aging models are considered the optimal approach for replicating the aging process of the human brain in neurodegenerative research. However, chronic biochemical defects such as cancer, hypertension, hyperlipidemia, and diabetes mellitus can obstruct the accuracy of parameters related to brain aging and result in the low survival rate.^22^ Administration of d-galactose has been utilized to better understand the brain aging process and to prevent other chronic biochemical disruptions.^23^ The cognitive function was used to assess the effects of d-galactose administration. The study of Guo et al. revealed that d-galactose administration increased oxidative stress and impaired cognitive function in mice.^24^ We found that d-galactose also caused a decrease in the NORT preference index in the A group. Furthermore, the D-gal + CUMS model had a significant influence on the NORT preference index in the AS group. However, the FAS group, which was treated with fish oil, was able to restore the working memory function as demonstrated by the NORT preference index.

The CUMS model was found to be in line with the environmental triggers of human depression.^16^ Previous studies have demonstrated that administering CUMS for 4–8 weeks can induce depressive-like symptoms in animals by consecutively exposing them to psychological stressors.^14,25–27^ In this study, the CUMS model was used followed by confirmation that blood glucose and MDA levels were elevated in week 16 (data not shown).^28^ The depressive symptoms were used to evaluate the effects of CUMS stimulation. The SPT detected physical expression of anhedonia and ensured that the CUMS stimulation had induced depression. Additionally, d-galactose administration (A group) did not cause anhedonia throughout the experiment. FST, which is more specific to depression-related pharmacology research, also had similar results.^29^ Beneficial effects on depressive-like symptoms were observed in the n-3 PUFA-treated group (FAS group), while no such effects were seen in the n-6 PUFA-treated group (CAS). Nevertheless, although we successfully induced the aged process and depressive-like symptoms simultaneously, this geriatric depression model is still in its early stages to accurately mimic late-onset depression. One important issue is the period during which we administered d-galactose. Previous studies revealed that the 4–10 week administration period can effectively induce oxidative stress. However, we did not observe significantly increased MDA levels in plasma until week 16. An article reported longer intervention periods than those of previous studies, such as our experiment.^30^ These findings showed that this model has some factors to be optimized.

Systemic oxidative stress induced by d-galactose is believed to activate the AGE/RAGE pathway, suppress anti-inflammatory pathways, and increase TNF-α levels, thus disrupting the blood–brain barrier (BBB) and causing eventual neurodegeneration.^31–34^ Our study found that AGE, MDA, and 8-OHdG (markers of DNA/RNA damage) were increased in the d-galactose administration group. CUMS stimulation and different diets had no effect on 8-OHdG and AGE. Previous studies indicated that depression was highly associated with colon disease, such as irritable bowel syndrome.^35,36^ Psychological stress was reported as resulting in decreasing the mucus thickness and disrupting the integrity of mucus. RNA sequencing analysis showed that stress shifts intestinal epithelial cells transcriptome toward elevated inflammation, ROS production, and antimicrobial defenses pathways.^37^ Thus, we were also interested in the effects of aging induction and CUMS stimulation on the colon. Accordingly, the D-gal administration could induce inflammation in the colon, while CUMS stimulation did not increase inflammation status. However, a high n-6 PUFA corn oil diet exacerbated the gut inflammation status, which indicated that high intake of n-6 PUFA was detrimental.

Similar results were observed in the central nervous system (CNS). D-gal-induced aging significantly elevated IL-1β level but not IL-6 and TNF-α levels. Furthermore, CUMS stimulation advanced increased TNF-α levels but not IL-1β or IL-6 levels. According to the results, the effects of aging and CUMS stimulation on different kinds of cytokines were worthy to discover. On the other hand, long-term consumption of low n-6/n-3 PUFAs has been suggested to reduce TNF-α and IL-6 levels.^38^ Several clinical trials have suggested that a high dietary intake of n-3 PUFAs can reduce the level of TNF-α and improve impaired cognition.^39,40^ Our study found that after 12 weeks of high fish oil diet, IL-1β levels were reduced in the hippocampus, and the corn oil diet with high n-6 PUFAs exacerbated the inflammation condition by increasing IL-6 and TNF-α levels. Thus, we infer that long-term n-3 PUFA intake reduces neuroinflammation and that high n-6 PUFA intake exacerbates neuroinflammation in the hippocampus, while the CUMS would worsen neuroinflammation but not oxidative stress in this model.

Chronic neuroinflammation has a wide-reaching effect on the CNS, including the integrity of the BBB, neuron survival, and neuroplasticity. This can inhibit neurogenesis and lead to neurodegeneration and the development of mental illness.^41–43^ Morphology of glial cells is an important factor in this mechanism. Shwe et al. showed that d-galactose administration reduces synaptic protein and neuroplasticity and increases the number of activated microglia (IBA1^+^ cells). This also results in decreased ramification of microglia and impaired cognitive function in Wistar rats.^21^ Another study revealed that the expression of CD68 in the hippocampus of aged rats (24 months old) was significantly increased and was associated with the impaired cognitive functions.^44^ In this study, we evaluated the inflammation status in different brain regions using several methods. In the hippocampus, we used IHC to detect activated microglia with high expression of IBA1 and CD68. We observed more IBA^+^ cells and CD68^+^ cell in the hippocampus of A, AS, and CAS groups compared to the C group. Neuroinflammation was partially relieved through imipramine treatment and fish oil intervention. Since microglial dysmorphology and elevated pro-inflammatory cytokines are associated with cognitive dysfunction,^45^ we suggested that the increasing IBA1 and CD68 in the hippocampus may be responsible for impaired cognitive functions. Nevertheless, similar outcomes were observed between A and AS groups, suggesting that d-galactose administration caused neuroinflammation and cognitive impairment, and the CUMS stimulation would not deteriorate them.

Tryptophan is one of the essential amino acids in humans. It is involved in the production of several neurotransmitters, including serotonin, melatonin, and kynurenine. 5-HIAA, the metabolite of serotonin, was considered as a crucial marker of pathophysiology of depression in clinical studies. Recently, meta-analyses have shown that the level of 5-HIAA in cerebrospinal fluid has no significant association with depression symptoms. This suggests that 5-HT and its metabolite 5-HIAA levels are influenced by age, gender, body height, severity of symptoms, analytics, and medication.^46–48^ In our study, we found that d-galactose plus CUMS stimulation (AS group) reduced serotonin levels, while fish oil diet and imipramine treatment increased serotonin in the hippocampus (FAS and MAS group). In contrast, d-galactose administration raised 5-HIAA levels, and CUMS stimulation had no effect on 5-HIAA. Our results indicate that the fish oil diet and imipramine improved serotonin levels and decreased 5-HIAA levels. Nevertheless, the reduced 5-HIAA levels were not associated with improved depressive-like symptoms. Previous studies proved that activated microglia participate in disrupting homeostasis of kynurenine pathway associated with major depressive disorder (MDD).^49^ LPS administration or chronic mild stress paradigm stimulation to male rodents increased inflammation in the periphery and induced depressive-like phenotypes, which resulted in the activated kynurenine pathway and increased the level of kynurenine.^50,51^d-galactose administration (A group) did not increase kynurenine and KYN/TRP ratios, but CUMS stimulation significantly raised them. This result could be strongly associated with the depressive-like symptoms induced by CUMS stimulation. However, the fish oil diet and imipramine had no effect on either of them, suggesting that fish oil might not alleviate depressive-like symptoms by inhibiting the kynurenine pathway. KA had some confusing experiment results. One study demonstrated that KA may exert a neuroprotective effect through antagonism of the *N*-methyl-d-aspartate receptor, protecting against the excitotoxic and apoptosis effect.^52^ Nevertheless, various recent articles had different results compared to the original study, which means the conclusion was controversial.^53^ In the present study, the KA level was increased in the AS groups. Although the potential effect of KA is still unknown, our result found that CUMS stimulation raised the KA level and caused depressive-like symptoms. A review article reported different MDD-related mechanisms of the balance of quinolinic acid (QA) and KA in the synaptic cleft between the peripheral nervous system and CNS.^49^ Our next goal is to elucidate the different QA/KA balances in peripheral nervous system and CNS under d-galactose plus CUMS stimulation.

In terms of neuroinflammation, the integrity of the BBB also plays an essential role in maintaining homeostasis in the CNS environment. An impaired BBB structure results in harmful external substances entering the CNS and causing neurodegeneration.^54^ In this study, we observed that ZO-1 expression in PFC was decreased by d-galactose administration, while occludin was decreased by d-galactose and CUMS stimulation. This result is inconclusive regarding the effects of d-galactose or stress on the tight junction. However, the disrupted tight junction results were responsible for IBA1 and GFAP expression. We observed that the GFAP expression was slightly reduced with the CUMS stimulation (not significant). Fish oil intervention was beneficial in our study, ameliorating GFAP and IBA1 overexpression induced by aging and CUMS stimulation. By contrast, the corn oil intervention did not recover the neuroinflammation. The GFAP expression has been demonstrated as a reliable marker presenting the astrocytes’ accumulation in aging individuals,^30^ but in patients with depression, it was still inconsistent. A study reported that MDD was associated with higher astrogliosis and reduced density of astrocytes in the prefrontal cortex.^55^ Another post-mortem study demonstrated that patients with MDD had decreased GFAP expression in the prefrontal cortex.^56^ Therefore, the effects of CUMS stimulation on the astrocytes need more evidence to conclude.

Some limitation of this study should be noticed. First, the NORT is just one type of cognitive function test to assess working memory. We did not perform other cognitive tests to rule out the different cognitive functions including learning and spatial memory. Future research should take this into account and include more tests. Second, d-galactose administration and CUMS stimulation had some inconsistent effects. Some parameters were affected by both aging and depression, but there was no synergistic effect, which means that CUMS did not deteriorate some results. Considering the outcomes of multiple neurobehavioral tests, there is a pressing need to develop a more fitting induced-aging depression model, one that effectively integrates both aging and depression factors. Future studies should include a group solely treated with CUMS stimulation to better understand the individual influences. Despite these limitations, this study was expected to provide a fundamental framework for the establishment of a much more reliable geriatric depression animal model induced by d-galactose and CUMS. This could potentially serve as a substitute for the natural aging model. Finally, when discussing the potential alteration of tryptophan catabolites and their effects on the neurobehaviors of rats, we did not assess neurotoxic QA metabolized by kynurenine. Future works should determine this compound to gain a more comprehensive understanding of tryptophan metabolism.

In conclusion, the geriatric depression model using D-gal administration and CUMS resulted in neuroinflammation and impaired BBB integrity in the hippocampus and PFC, which led to alteration of the tryptophan metabolism and abnormal neurobehaviors. Supplementing the rats with fish oil containing n-3 PUFAs for 12 weeks alleviated neuroinflammation and psychiatric symptoms.

## Supporting Information Available

Ingredients and nutrient compositions of the animal diet and analytes used for MRM conditions in positive ionization mode (PDF)

## Abbreviations

AST: aspartate aminotransferase
ALT: alanine aminotransferase
TC: total cholesterol
TG: triglyceride
BUN: blood urine nitrogen
LDL-c: low-density lipoprotein cholesterol
HDL-c: high-density lipoprotein cholesterol
3-HK: 3-hydroxykynurenine
5-HIAA: 5-hydroxyindoleacetic acid
5-HT: 5-hydroxytryptamine
8-OHdG: 8-hydroxy-2′-deoxyguanosine
AA: arachidonic acid
AGEs: advanced glycation end products
CUMS: chronic unpredictable mild stress
D-gal: d-galactose
DHA: docosahexaenoic acid
EPA: eicosapentaenoic acid
FST: forced swim test
GFAP: glial fibrillary acidic protein
IBA1: ionized calcium-binding adapter molecule 1
IL-1β: interleukin 1 beta
IL-6: interleukin-6
IL-10: interleukin-10
iNOS: inducible nitric oxide synthase
KA: kynurenic acid
MDA: malondialdehyde
NORT: novel object recognition test
PUFA: polyunsaturated fatty acid
TBARS: 2-thiobarbituric acid reactive substances
TNFα: tumor necrosis factor α
SPT: sucrose preference test
WHO: World Health Organization
ZO-1: zonula occludens-1
