# Supplementary material for: Attenuation of Chronic Stress-Induced Depressive-like Symptoms by Fish Oil via Alleviating Neuroinflammation and Impaired Tryptophan Metabolism in Aging Rats
Source: J Agric Food Chem. 2023 Sep 28;71(40):14550–61. doi: 10.1021/acs.jafc.3c01784 (PMC10915802; doi:10.1021/acs.jafc.3c01784)
Supplement: Supplementary file 1 — jf3c01784_si_001.pdf [file jf3c01784_si_001.pdf]

Supporting Information

Attenuation of chronic stress-induced depressive-like symptoms by fish oil via alleviating neuroinflammation and impaired tryptophan metabolism in aging rats

Te-Hsuan Tung,[a] Wen-De Lai,[a] Hsiu-Chuan Lee,[a] Kuan-Pin Su,[b] Binar Panunggal,[a,c] and Shih-Yi Huang, [a,d,e*]

[a] School of Nutrition and Health Sciences, Taipei Medical University, Taipei 110301, Taiwan

[b] Department of Psychiatry & Mind-Body Interface Laboratory (MBI-Lab), China Medical University Hospital, Taichung, Taiwan; College of Medicine, China Medical University, Taichung 404018, Taiwan

[c] Department of Nutrition Science, Faculty of Medicine, Diponegoro University, Semarang, Indonesia; Center of Nutrition Research, Diponegoro University, Semarang, Indonesia

[d] Graduate Institute of Metabolism and Obesity Sciences, Taipei Medical University, Taipei 110301, Taiwan

[e] Nutrition Research Centre, Taipei Medical University Hospital, Taipei 110301, Taiwan

* Corresponding author: Shih-Yi Huang (sihuang@tmu.edu.tw)

ORCID iD: 0000-0003-2914-5513 (SY Huang)

Contents

Supplementary table

Supplementary table S1: Ingredients and nutrients composition of the animal diets.

Supplementary table S2: List of analytes used for multiple reaction monitoring (MRM) conditions in positive ionization mode.

Supplementary table S1: Ingredients and nutrients composition of the animal diets [a]

Ingredients (g/kg dry matter)	Group		
	C, A, AS, MAS	FAS	CAS
Casein	140.00	140.00	140.00
L-Cystine	1.80	1.80	1.80
Corn starch	465.69	465.69	465.69
Dextrin	155.00	155.00	155.00
Sucrose	100.00	100.00	100.00
Cellulose	50.00	50.00	50.00
Soybean oil	40.00	20.00	20.00
Corn oil	0	0	20.00
LA	0	0	11.2
Fish oil	0	20.00	0
Eicosapentaenoic acid (EPA)	0	3.60	0
Docosahexaenoic acid (DHA)	0	2.40	0
AIN-93M Mineral mix	35.00	35.00	35.00
AIN-93M Vitamin mix	10.00	10.00	10.00
Choline bitartrate	2.50	2.50	2.50
t-butylhydroquinone	0.01	0.01	0.01
**Fatty acid profiles (%)**			
C 14:0	1.42	5.63	1.23
C 16:0	13.05	18.02	10.41
C 16:1 n-7	0.07	0.04	0.03
C 18:0	4.87	7.97	6.22
C 18:1 n-9	17.79	9.95	19.01
C 18:2 n-6	52.71	28.74	59.52
C 18:3 n-3	9.88	6.19	3.26
C 20:1 n-9	N.D.	0.27	N.D.
C 20:2 n-6	N.D.	0.38	N.D.
C 20:4 n-6	0.01	4.08	0.01
C 20:5 n-3	N.D.	9.63	N.D.
C 22:5 n-3	0.18	1.46	0.08
C 22:6 n-3	N.D.	5.98	N.D.
n-6/n-3 ratio	5.24	1.30	17.82

[a] Values are presented as grams or percentages.

Supplementary table S2: List of analytes used for multiple reaction monitoring (MRM) conditions in positive ionization mode.

No.	Analytes	Precursor ion (m/z)	Product ion (m/z)
1	kynurenine	209.0	192.0
2	kynurenic acid	189.9	143.9
3	3-hydroxykynurenine	225.0	109.8
4	serotonin	177.0	160.0
5	tryptophan	204.8	188.0
6	5-hydroxyindoleacetic acid	192.0	146.0
Internal Standard	acetaminophen	152.0	109.9
